# Exploring Mutuality in Nurse–Care Recipient Relationships in a Primary Care Setting: A Qualitative Descriptive Study

**DOI:** 10.1155/jonm/1520015

**Published:** 2026-05-06

**Authors:** Erica Busca, Isabella Santomauro, Silvia Caristia, Angela Durante, Federica Manservisi, Simona Milani, Ines Basso, Erika Bassi, Alberto Dal Molin

**Affiliations:** ^1^ Department of Translational Medicine, University of Piemonte Orientale, Via Solaroli 17, Novara, 28100, Italy, unipmn.it; ^2^ Department for Sustainable Research and Ecological Transition, University of Piemonte Orientale, P.za Sant’Eusebio 5, Vercelli, 13100, Italy, unipmn.it; ^3^ Tuscany Foundation “G. Monasterio,”, Via Giuseppe Moruzzi 1, Pisa, 56124, Italy; ^4^ Health Science Interdisciplinary Center, Scuola Superiore Sant’Anna, Piazza Martiri Della Libertà 33, Pisa, 56127, Italy, sssup.it; ^5^ Local Health Authority of Biella, Via Dei Ponderanesi 2, Ponderano, 13875, Italy

**Keywords:** continuity of patient care, home care services, nurse–patient relations, qualitative research, trust

## Abstract

**Background:**

In primary care (PC), nurses ensure continuity and person‐centered care, fostering long‐term relationships with patients and caregivers. How mutuality, a relational process closely linked to patient‐centered communication, develops in this context remains underexplored.

**Aims:**

To describe the characteristics of mutuality and explore how it develops in the relationships between nurses and care recipients in a PC setting.

**Method:**

A qualitative descriptive study was conducted in a PC setting. Participants included 14 nurses trained in Family and Community Nursing and 27 care recipients (patients and family caregivers). Data were collected between May and July 2024 through semistructured interviews. A total of 28 interviews were conducted, including both individual and dyadic interviews with patients and caregivers. Data were analyzed using framework analysis, guided by a conceptual framework on mutuality in the nurse–patient relationship. The framework focused on influencing factors, the mutuality process, and outcomes.

**Results:**

Findings from 28 interviews revealed that mutuality was influenced by both organizational and personal factors. Nursing care delivery models that ensured continuity of care and nurse autonomy supported trust, stable, and long‐term relationships. The process of mutuality unfolded progressively through three dimensions: (1) building and going beyond the formal encounter, (2) being a consistent point of reference, and (3) deciding and sharing care. Mutuality emerged as a co‐constructed relationship, shaped by clear communication, trust, and shared responsibilities, with informal caregivers recognized as integral participants. Outcomes included strengthened professional identity and satisfaction for nurses and improved emotional well‐being, self‐care, and self‐efficacy for care recipients.

**Conclusion:**

In PC, mutuality transforms task‐oriented interactions into meaningful, trust‐based relationships, enhancing patient‐centered communication and shared decision‐making. Care continuity and autonomy, combined with investment in relational skills, are essential to sustain mutuality, improve communication, and enhance care quality.

## 1. Introduction

Population aging represents one of the most significant global challenges [1]. In Europe, the proportion of people aged 65 and over is projected to rise from 21% to 30% by 2050 [2]. According to the Organization for Economic Co‐operation and Development [2], only two‐thirds of these years are lived in good health; the remaining years are often marked by disability and dependency, necessitating domiciliary care and the support of an informal caregiver, where available [2]. The high prevalence of such conditions has underscored the urgency of adopting integrated, community‐based models of care capable of addressing complex and long‐term health and social needs [1]. In response to these challenges, several countries have strengthened primary care (PC) systems to promote continuity, accessibility, and coordination of care. In Italy, Ministerial Decree (MD) 77/2022 [3] introduced a new structure for community‐based healthcare, emphasizing the integration of health and social services and the provision of care closer to patients’ homes. Within this new structure, nurses play a central role in strengthening the PC network and supporting individuals within their family and social context [3]. In PC settings, nurses establish ongoing relationships with patients and informal caregivers, contribute to personalized care planning, and support the achievement of shared health goals [4–6].

The nurse–patient relationship has been conceptualized as a complex and dynamic relational process grounded in mutual respect, engagement, and interaction [7, 8]. Within these relationships, mutuality represents a key relational dimension. Mutuality has been described as a process characterized by interdependence, collaboration [9], and reciprocity [10], through which care moves beyond task‐oriented interactions toward a more person‐centered approach. In this perspective, both nurses and care recipients (CRs) actively contribute to the relationship and to the definition of care goals, fostering shared decision‐making and relational equity [11]. Previous studies have shown that such relationships can lead to beneficial outcomes, including improved quality of life [12, 13], enhanced self‐care [14, 15], and reduced levels of stress [16] and depressive symptoms [12, 17] in both patients and caregivers.

Although mutuality in nurse–patient relationships has been explored in both inpatient and outpatient settings [11, 18], and a small number of studies have adopted a dyadic perspective [19], there has been limited attention to how mutuality develops over time, particularly in PC settings. PC setting provides a unique context for the development of mutuality. Unlike hospital settings, which are often characterized by episodic and time‐limited interactions, PC enables continuity of care, repeated encounters over time, and the possibility to engage with patients within their home and family environment. These conditions support the gradual development of trust, shared understanding, and collaborative decision‐making, involving not only patients but also informal caregivers. Despite these characteristics, there is still a lack of qualitative studies exploring how mutuality develops in PC settings between nurses and CRs, particularly from both perspectives. Understanding this process is essential to inform models of care that support relational continuity and patient‐centered communication.

The aim of this study is to describe the characteristics of mutual relationships between nurses and CRs and to explore how mutuality develops within a PC setting.

## 2. Methods

### 2.1. Study Design

A qualitative descriptive design was used to gain a rich, contextualized understanding of participants’ experiences [20]. This approach is particularly appropriate when investigating poorly understood phenomena, as it allows for the presentation of findings using language that closely mirrors participants’ own words and meanings [21, 22].

The study was reported in accordance with the Standards for Reporting Qualitative Research (SRQR) [23].

### 2.2. Theoretical Framework

The study was guided by the conceptual framework for mutuality in the relationship between nurses and patients proposed by Cilluffo et al. [11]. This framework identifies potential factors influencing the development of mutuality, the mutuality process, and potential outcomes for both nurses and patients.

It informed the development of the interview guide and structured the deductive coding during data analysis.

### 2.3. Setting and Participants

The study was conducted in a Local Health Authority (LHA) in Northern Italy and involved two distinct participant groups: nurses employed in a PC setting and CRs. Eligible nurses were recruited from seven nursing teams, each responsible for a defined geographical area. Inclusion criteria for nurses were completion of postgraduate training in Family and Community Nursing and active employment in a PC setting. Inclusion criteria for patients were as follows: age ≥ 65 years, receiving ongoing care from a nurse for at least one year or having received ≥ 3 visits, and the ability to share detailed descriptions of their experiences. The criterion of ≥ 1 year of care or ≥ 3 visits was used to ensure that participants had sufficient exposure to the nurse–patient relationship. Inclusion criteria for caregivers were being identified as a family member providing support with activities of daily living to a person receiving domiciliary care, regardless of the time dedicated to caregiving. CRs were identified through purposive sampling and included both older adult patients and their informal caregivers. Nurses provided an initial list of potentially eligible participants based on the predefined inclusion criteria.

Identified CRs were subsequently visited at home by a nurse researcher (EB), who invited them to participate in the study. Nurses were not involved in the consent process or in CRs’ decision to participate. During the home visit, participants were asked to sign the informed consent form and then provided their availability to schedule an individual interview at a convenient time and date.

### 2.4. Data Collection

Data were collected between May and July 2024 through semistructured interviews conducted by the nurse researcher, an expert in qualitative health research (EB). Interviews took place at a location convenient for participants, either their home or workplace, and lasted approximately 40 min. The interviews aimed to explore participants’ experiences and perceptions regarding the development of mutuality within the nurse–CR relationship in the PC setting (Table 1). The interview guide was refined through discussion within the research team. Interviews were conducted in a flexible manner, allowing participants to elaborate on their experiences and introduce relevant topics. They were carried out either individually or jointly (patient–caregiver dyads), depending on participants’ preferences, and took place face‐to‐face with audio recording. Following each interview, field notes were maintained to document contextual details, such as the physical setting, and observations related to the participants’ level of engagement and emotional responses. Demographic data were also collected.

**TABLE 1:** Sample questions used in the semistructured interviews.

Questions asked to nurses	Questions asked to care recipients
“What factors influence the development of a positive relationship with the patient and caregiver?”	“What helped or made it difficult to build a good relationship with the nurse?”
“How do you construct a relationship with the patient and caregiver?”	“Can you describe how your relationship with the nurse began and how it has developed over time?”
“How does this relationship affect the family? Does it influence your professional or personal experience?”	“Do you think your relationship with the nurse has affected your care experience?

### 2.5. Data Analysis

A framework analysis approach was used, following the stages outlined by Gale et al. [24]. Interviews were transcribed verbatim using NVivo transcription software, anonymized, and checked for accuracy by the research team prior to analysis. Two researchers (EB and FM) independently familiarized themselves with the transcripts through repeated reading. A primarily deductive coding approach was used, guided by the conceptual framework [11], while remaining open to additional codes emerging from the data. Initial codes were developed and discussed within the research team. Code saturation was monitored throughout the analytical process until no new meaningful codes emerged [25]. This allowed for the development of a working analytical framework, which was subsequently applied systematically to all transcripts. The coded data were then charted into a matrix, where summaries of relevant data were entered into cells organized by case (rows) and by code (columns). This structure allowed for comparison both within and across participants.

### 2.6. Rigor

Rigor was ensured through strategies aligned with established criteria for trustworthiness in qualitative research [26]. Credibility was supported through data source triangulation, including nurses, patients, and caregivers, as well as through peer debriefing with colleagues. Field notes were used to contextualize the interviews and to support data interpretation, contributing to the credibility of the findings. Dependability and confirmability were enhanced through independent coding and regular team meetings to reflect on emerging findings. Transferability was promoted by providing detailed contextual information and participant characteristics.

### 2.7. Ethical Considerations

The study received ethical approval from the local ethics committee (Approval Number: CE 104/2024). Informed consent was obtained from all participants prior to data collection. To ensure confidentiality and anonymity, each interview was assigned a unique alphanumeric code, and all identifying information was removed from transcripts.

## 3. Findings

Fourteen nurses with postgraduate training in Family and Community Nursing and 27 CRs participated in the study. Fifteen interviews were conducted with CRs, including patients and informal caregivers. These included four interviews with patients alone, eight with patient–caregiver dyads, and three with caregivers alone. The characteristics of the sample are reported in Table 2.

**TABLE 2:** Characteristics of participants.

	**FCNs (*N* = 14)**	

Female, *n* (%)	12 (85.7)	
Age, mean (±SD) range [min–max]	47.6 (±6.7) [36–56]	
Overall work experience (year), mean (±SD) range [min–max]	22.9 (±7.2) [13–35]	
Work experience as FCNs (year), mean (±SD) range [min–max]	3.1 (±2.8) [0.8–8]	

	**Care recipients (*N* = 27)**	
	**Caregivers (*N* = 11)**	**Patients (*N* = 16)**

Female, *n* (%)	10 (91)	6 (35.3)
Age, mean (±SD) range [min–max]	69.2 (±14.2) [49–89]	81.8 (±9.7) [71–96]
Relationship with patient (for caregivers), *n* (%)		
Wife	7 (63.6)	NA
Son	1 (9.1)	NA
Daughter	3 (27.3)	NA
Time dedicated to caregiving (daily), mean (±SD) range [min–max]	18.9 (±8.2) [2–24]	NA
Length of nursing care (years), mean (±SD)^∗^	2.1 (±2.1)	
Number of nursing interventions provided to patients, *n* (%)		
≤ 4	NA	13 (81.3)
> 4	NA	3 (18.7)
Main types of nursing interventions provided to patients, *n* (%)		
Therapeutic education	NA	16 (100)
Wound care	NA	15 (93.6)
Blood sampling	NA	15 (93.6)
Clinical monitoring	NA	8 (50)
Injection administration	NA	7 (43.6)

Abbreviations: FCNs, Family and Community Nurses; NA, not applicable.

^∗^Missing.

Findings are presented according to the three main constructs from the conceptual framework on mutuality [11]: (1) influencing factors, (2) mutuality process, and (3) outcomes. Table 3 provides a comprehensive summary of the constructs, subconstructs, categories, and related codes.

**TABLE 3:** Summary of constructs, subconstructs, categories, and related codes based on the mutuality conceptual framework.

Construct	Subconstruct	Categories	Codes
1. Influencing factors	Nurses’ influencing factors	Professional experiences	Prior experience in hospital settings Experience gained in primary care settings
		Organizational model	The home as a care setting Continuity of care One‐to‐one care relationship Functional need triggering the relationship
		Personal characteristics	Empathic interaction style
	Patients’ influencing factors	Personal experiences	Knowing the nurse beforehand

2. Mutuality process		Building and going beyond	Awareness of needing the nurse Initial presentation and role clarification Setting boundaries Understanding the patient and their family context Trusting and letting oneself be guided Evolution from formality to familiarity
		Being a reference	Consistent presence Being a family member Point of reference for practical issues Emotional support Reference figure in communication with the GP Established a routine that creates expectation
		Deciding and sharing care	Offering tailored solutions Adaptation to patient’s readiness Step‐by‐step sharing of the care pathway Recognition and caregivers activation

3. Outcomes	Nurse‐related outcomes	Job satisfaction	Ongoing relational connection with caregivers Decision‐making autonomy Achievement of health‐related goals
		Burden	Emotional engagement (beyond professional boundaries) Communicative strain Coming to terms with the potential failure of care
		Competence	Patient recognition of technical care competence Adaptive communicative competence
	Patient‐related outcomes	Self‐care	Development of self‐care skills Family engagement
		Self‐efficacy	Increased self‐efficacy Low perceived self‐efficacy in older caregivers
		Satisfaction	Safety and relief Appreciation of timeliness and continuity of care
		Quality of life	Resumption of daily and meaningful activities Emotional well‐being
		Clinical outcomes	Wound improvement
		Emergency access, hospital admission	Reduction of inappropriate hospital access Facilitation of access to healthcare services and social services

### 3.1. Influencing Factors

#### 3.1.1. Nurses’ Influencing Factors

Nurses reported that prior work experience, both in hospital and PC settings, strengthened their technical skills and their ability to understand patients’ life contexts. These experiences also enabled them to guide patients and families in navigating health services (e.g., the prosthetics service for assistive device requests). Alongside their professional background, nurses highlighted the value of an empathetic approach, seen as essential for identifying CRs’ needs and fostering mutual understanding. As one caregiver stated: “*She (the nurse) came to our home to meet us and told us she would be our reference nurse. We found her very kind, very skilled—she wanted to know our whole story*” (CR_3).

Another factor that positively influenced mutuality was the organizational model through which nursing care was delivered. In this model, each patient was assigned to a reference nurse who took responsibility for the care pathway. According to nurses, this ensured continuity of care, starting with the GP’s referral and continuing over time with a level of intensity that adapted to the patient’s clinical condition. From the patients’ perspective, having a reference nurse meant establishing a continuous relationship, characterized by consistent contact and regular scheduled visits: *“I feel reassured because I know she (the nurse) comes on Tuesdays and Fridays for the injections, and I already know what she will do*” (CR_6). This continuity supported the development of trust and relational stability.

This organizational model also shaped how nursing care was initiated, which typically began in response to a task‐oriented need. CRs described the fulfillment of urgent or functional request, such as wound dressing, blood draw, or catheter change, as the initial trigger for the relationship. However, nurses described how entering the home to carry out a procedure often provided the first opportunity to build a relationship and gain a deeper understanding of the patients, their caregivers, and the home environment. This transition from task‐oriented care to relational engagement reflects the early development of mutuality. As one nurse explained: *“The procedure becomes your way in. If the person needs that at that moment, the family expects exactly that. But once you’re in the home and show empathy, it’s no longer just about the procedure. You start to get to know them better, to understand their needs… And in the meantime, we end up doing something more than just the task*” (N_12).

#### 3.1.2. Patients’ Influencing Factors

Patients reported that having had previous contact with the nurse currently involved in their care facilitated the development of a positive relationship. In some cases, this familiarity derived from long‐standing community ties, with patients having known the nurse since childhood. In others, familiarity was rooted in CRs’ prior experiences with the same nurse, who had previously provided care to a family member. These experiences, as perceived and recalled by a caregiver, contributed to shaping initial trust and expectations toward the nurse: *“I had a wonderful experience with my parents—this was about 15 years ago—my mother had a severe stroke, and the nurse took care of her perfectly”* (CR_10).

### 3.2. Mutuality Process

#### 3.2.1. Building Trust and Going Beyond

The initial encounter played a crucial role, as it often took place in a naturally unbalanced dynamic; patients or caregivers were typically in a position of need and partial dependence. According to participants, this first interaction laid the foundation for the mutual relationship: on one side, patients expressed their concerns and expectations; on the other, nurses clarified their role and scope of practice, while also seeking to establish clear relational boundaries from the outset. As one nurse explained: “*When you begin to establish a relationship, you need to clearly present who you are and what you do […] Sometimes patients have expectations. Some say, ‘let’s get the nurse for this, this, and this…’ and at first it’s difficult, because the patient and the family need to understand what you’re there to do*” (N_5).

The first encounter was also described as a key moment to understand the patient beyond their clinical condition, including home context and significant relationships: “*During the first visit, we go to get to know the patient. You may know their health issues, but you don’t know the person—where they live, what surrounds them, what kind of relationships they have with their family*” (N_5).

Following this initial phase, the relationship gradually developed through repeated interactions over time. CRs described a relationship built on trust, which fostered an attitude of openness and a conscious willingness to rely on the nurse’s guidance. One patient explained that the alignment between what the nurse had said and how things actually turned out helped reinforce this trust: “*What they told me before starting treatment always turned out to be true, so I gained even more trust*” (CR_12).

As trust developed, the nature of the interaction also changed: what began as a formal, task‐oriented relationship gradually shifted toward a more spontaneous and familiar dynamic. Participants described small rituals, such as sharing coffee, and a more informal tone of communication, as signs of increasing relational closeness. This shift reflects the progressive development of reciprocity within the relationship. As one nurse noted: *“There are changes, because at the beginning everything is very formal. Then they start asking, ‘Can I speak to you informally?’ I used to find it difficult, but then I realized they feel uneasy, they think you’re trying to keep your distance*” (N_7).

#### 3.2.2. Being a Point of Reference

Nurses were perceived by CRs as a point of reference due to their ongoing and consistent presence over time, as well as their ability to handle practical needs, such as supplying materials (e.g., gauze and enteral feeding bags). This presence was not limited to the home visit but extended to a broader and continuous professional engagement, including accessibility and proactive support. According to CRs, this engagement was reflected in a proactive and responsive approach: the nurse acted as a mediator with the GP, took initiative when new needs or concerns arose, and remained accessible throughout the day. This combination of actions and attitudes positioned the nurse as the first point of contact for CRs. As one patient stated: *“Of course, if our nurse isn’t available, someone else will come—but he’s still our point of reference. He’s always there if we need something: ‘hello, here I am’”* (CR_2).

Over time, this ongoing presence contributed to the development of a stable and reliable relationship. Some CRs described the nurse as “part of the family,” reflecting a growing sense of belonging and the emergence of relational expectations. As one caregiver stated: *“She’s (the nurse) really like one of the family. She sees when something isn’t right and always tries to sort it out. She’s just like someone from the household—really part of it. [Name of the patient] always asks her, ‘When are you coming back to see me?’”* (CR_6).

#### 3.2.3. Deciding and Sharing Care

Nurses highlighted that a key condition for building a shared care pathway was the ability to tailor their approach to the patient’s level of engagement and readiness. They described how essential it was to recognize and respect individual limits and preferences. This often involved negotiating small adjustments, so that care goals were experienced as mutually defined and realistically achievable.

In parallel, nurses recognized the active role of caregivers in the care process. This supported a realistic form of collaboration, where each person contributed according to their own possibilities and resources. Nurses described how explaining each step of care enabled patients and caregivers to feel more confident and progressively more autonomous. On this basis, a gradual process of shared care planning developed, guided by clear and anticipatory communication: *“If we anticipate and say ‘first we’ll do this, then this, and then we’ll end with that,’ they feel reassured and say ‘okay’ […] If you explain things well and they understand, then they are able to continue even on their own”* (N_7).

### 3.3. Outcomes

#### 3.3.1. Nurse‐Related Outcomes

Nurses described that building a mutual relationship brought a sense of satisfaction across several levels, clinical, relational, and related to autonomy in decision‐making. On the clinical level, they experienced gratification from the continuity of care and the opportunity to observe the tangible results of their work over time. As one nurse explained: *“What I see as different compared to the hospital setting is that I truly reap the fruits of my work. You’re the one who follows that person all the time, and so you can see whether the wound is healing. It’s rewarding, if things go well. […] Here, even for small things, you hear ‘thank you.’ It’s rewarding—both personally and professionally”* (N_10). On a relational level, satisfaction was linked to recognition from CRs, some of whom remained in contact even after the care pathway ended. Nurses interpreted this continued contact as validation of both their professional role and the relationship they had built. Finally, from a decision‐making perspective, mutuality reinforced nurses’ sense of autonomy and competence in the care process.

However, alongside these positive outcomes, the emotional effort involved in building a mutual relationship also led to feelings of fatigue and frustration. Some nurses described the challenge of coming to terms with the potential failure of care. This was especially evident in situations involving difficult communication, where relationships were characterized by resistance and a lack of mutual understanding. One nurse shared her frustration in trying to help a family accept the care recommendations: *“I found it hard to make myself understood: I wasn’t saying to reposition her father every 2 hours just for the sake of it. It’s a good practice to prevent pressure injuries—he needed it. I struggled with this family, because they wouldn’t accept the situation. […] The daughter kept asking other people—second, third opinions. I felt like I was missing something”* (N_11).

#### 3.3.2. Patient‐Related Outcomes

CRs revealed several positive outcomes. One of the most significant was the progressive development of self‐care abilities, often supported by educational intervention. Nurses described situations in which families, initially resistant, gradually became able to self‐care: *“At first, the wife didn’t want to connect the parenteral nutrition bag. She said, ‘I won’t do it.” Then I started with the daughter, I explained the whole procedure, and she learned how to do it. And over time, the mother, just by being there, began preparing the parental nutrition bag with me. In the end, they knew more than I did—and they were able to manage everything on their own”* (N_7). However, not all caregivers felt confident managing devices independently, particularly older caregivers, who often expressed a low sense of self‐efficacy.

The nurse’s presence also had a direct impact on patients’ emotional well‐being. CRs described feelings of relief, safety, and, in some cases, an improvement in quality of life through the resumption of meaningful daily activities, such as working in the garden. One caregiver, for example, spoke of how the nurse’s presence marked a real turning point in her life: *“Since she arrived, everything has changed for me… I feel good—before, I felt a bit lost”* (CR_9).

From a clinical perspective, the mutual relationship translated into tangible health benefits, such as the healing of skin wounds, which was frequently mentioned by participants. Another outcome, reported by CRs, was a perceived reduction in inappropriate hospital visits. The nurse’s consistent presence made it possible to identify early signs of deterioration, manage problems at home, and provide timely support, thus avoiding emergency department visits for situations that could be handled locally. As one caregiver shared: *“The things she (nurse) does—we wouldn’t be able to do them ourselves. Thanks to her, so many times we’re able to manage things at home—so we don’t have to go to the hospital”* (CR_12).

## 4. Discussion

This study explored mutuality in the relationship between nurses and CRs within a PC setting, contributing to and expanding international literature. Most studies on mutuality have focused on hospital settings [18], where time constraints and organizational routines may limit relational development [27]. In contrast, PC offers conditions, such as continuity of care, that allow mutuality to evolve over time as a co‐constructed and trust‐based process. The findings, consistent with the conceptual framework proposed by Cilluffo et al. [11], are articulated across three constructs that reflect the evolving nature of mutuality: the factors influencing the relationship, the process through which mutuality develops, and the outcomes.

The study findings highlight the presence of mutuality in nurse–CR relationships, as well as the key characteristics that define them. The relationship between nurses and patients is a fundamental component of high‐quality nursing care. It is often referred to as “therapeutic” because of the purpose it serves within a complex network of interprofessional, family, and patient relationships [28]. Although some authors question whether true mutuality can be achieved given the inherent asymmetry of the nurse–patient relationship [10], participants described how mutuality can emerge in PC when nurses adopt a person‐centered approach that recognizes patients as active agents with values, expectations, and competencies. This strengthens the therapeutic dimension of the relationship.

In PC, the nurse–CR relationship often begins in response to a task‐oriented need, such as a wound dressing, blood draw, or catheter change. Participants described how these practical tasks served as entry points for initiating the relationship and establishing early recognition of the nurse’s professional role. The fact that nurses are perceived by CRs as a “point of reference” can be seen as a concrete expression of professional validation, one that extends beyond task execution and embeds the nurse within the family’s relational network. This recognition is strengthened through the continuity of care typical of domiciliary care services, which in turn fosters mutual trust [29]. As a result, the process of mutuality ultimately leads to reciprocal recognition, in which both parties no longer identify solely with their respective roles (professional/user), but begin to perceive each other as individuals engaged in a meaningful relationship [10]. From this perspective, as participants highlighted, mutuality can be understood as an authentic bond that may persist beyond the formal conclusion of the care pathway.

Finally, the study findings reveal an initial shift from a patient‐centered care model toward a more family‐centered approach. As described by participants, this transition is reflected in the progressive involvement of caregivers in care activities and decision‐making processes, supporting their role as partners in the care pathway, in line with family‐centered care models [30].

However, in the Italian context, this model remains underdeveloped due to the lack of formal institutional recognition of the caregiver as a legitimate recipient of care. This limitation has also been highlighted by Parmar et al. [31], who note that, although caregiver well‐being is closely linked to that of the patient, the healthcare system tends not to formally acknowledge caregivers’ needs, thereby limiting effective joint care management.

This study has some limitations. The use of purposive sampling based on nurses’ referrals may have introduced a positive bias, as participants with more stable or positively perceived relationships may have been more likely to be included, potentially limiting the exploration of more complex or challenging experiences of mutuality. In addition, the study was conducted in a single LHA in Northern Italy, within a context‐specific nursing care model, which may limit the transferability of the findings to other primary care settings with different organizational structures and levels of nursing autonomy. Finally, all participants were aged 65 years or older. Although this reflects the typical population receiving domiciliary care, it may limit the applicability of the findings to younger populations.

## 5. Conclusion

This study described the characteristics of mutuality in the nurse–CR relationships within a PC setting. Mutuality emerged through a process of reciprocal recognition, facilitated by relational continuity and a climate of trust. Participants described how trust developed over time through consistent interactions and alignment between communication and care practices. As a result, mutuality was associated with improved emotional well‐being, self‐care, and satisfaction for CRs, as well as professional fulfillment for nurses.

### 5.1. Recommendations

The study’s findings underscore how the relational dimension of nursing care shapes the entire care process. Mutuality is an essential component of the nurse–CR relationship, facilitated by organizational models that promote continuity, proximity, and personalized care. To support and strengthen mutuality, healthcare organizations should promote care models such as primary nursing [32], which enable continuity through the assignment of a reference nurse responsible for coordinating care over time and across settings. In addition, organizations should implement targeted training programs focused on communication and relational skills, including negotiation of care goals, active involvement of caregivers, and management of long‐term relationships.

## Author Contributions

Conceptualization: Erica Busca, Angela Durante, Erika Bassi, and Alberto Dal Molin. Investigation: Erica Busca. Methodology: Erica Busca, Erika Bassi, Angela Durante, Silvia Caristia, Federica Manservisi, and Alberto Dal Molin. Writing–original draft: Isabella Santomauro and Erica Busca. Writing–review and editing: Erica Busca, Erika Bassi, Ines Basso, Isabella Santomauro, Federica Manservisi, Angela Durante, Simona Milani, and Alberto Dal Molin. Supervision: Alberto Dal Molin. Project administration: Erica Busca, Erika Bassi, Simona Milani, and Alberto Dal Molin. Funding acquisition: Alberto Dal Molin.

## Funding

This study is part of the AGE‐IT national project, which has received funding from the MUR‐M4C2 1.3 of PNRR funded by the European Union, NextGenerationEU (Grant agreement no. PE_00000015).

Open access publishing was facilitated by Universita degli Studi del Piemonte Orientale Amedeo Avogadro, as part of the Wiley–CRUI‐CARE agreement.

## Disclosure

All authors have read and agreed to the published version of the manuscript.

## Conflicts of Interest

The authors declare no conflicts of interest.

## Data Availability

The data that support the findings of this study are available from the corresponding author upon reasonable request.

## References

[1] World Health Organization , Integrated Care for Older People (ICOPE): realigningRprimary hePlth carH to reCpond toRpopulationPageing 201A, https://iris.who.int/bitstream/handle/10665/326295/WHO-HIS-SDS-2018.44-eng.pdf?sequence=1.

[2] Oecd/European Commission , Health at a Glance: Europe 2024: State of Health in the EU Cycle, 2024, OECD Publishing, Paris, 10.1787/b3704e14-en.

[3] Ministero della Salute , Decreto 23 Maggio 2022, n.77. [Regulation Defining Models and Standards for the Development of Territorial Care Within the National Health Service 2022], https://www.gazzettaufficiale.it/eli/id/2022/06/22/22G00085/SG.

[4] McCrory V. , An Overview of the Role of the District Nurse Caring for Individuals with Complex Needs, British Journal of Community Nursing. (2019) 24, no. 1, 20–26, 10.12968/bjcn.2019.24.1.20, 2-s2.0-85059226748. 30589587

[5] Bolton R. E. , Bokhour B. G. , Hogan T. P. , Luger T. M. , Ruben M. , and Fix G. M. , Integrating Personalized Care Planning into Primary Care: a Multiple-Case Study of Early Adopting Patient-Centered Medical Homes, Journal of General Internal Medicine. (2020) 35, no. 2, 428–436, 10.1007/s11606-019-05418-4. 31650401 PMC7018887

[6] Ferraris G. , Dang S. , Woodford J. , and Hagedoorn M. , Dyadic Interdependence in Non-spousal Caregiving Dyads’ Wellbeing: A Systematic Review, Frontiers in Psychology. (2022) 13, 10.3389/fpsyg.2022.882389. PMC9102382 35572327

[7] Allande-Cussó R. , Fernández-García E. , and Porcel-Gálvez A. M. , Defining and Characterising the nurse–patient Relationship: a Concept Analysis, Nursing Ethics. (2022) 29, no. 2, 462–484, 10.1177/09697330211046651. 34879785

[8] Xue W. and Heffernan C. , Therapeutic Communication Within the nurse–patient Relationship: a Concept Analysis, International Journal of Nursing Practice. (2021) 27, no. 6, 10.1111/ijn.12938. 33949044

[9] Jeon Y. , Shaping Mutuality: Nurse−Family Caregiver Interactions in Caring for Older People with Depression, International Journal of Mental Health Nursing. (2004) 13, no. 2, 126–134, 10.1111/j.1440-0979.2004.0312.x, 2-s2.0-6944241661. 15318907

[10] Brown B. J. , Mutuality in Health Care: Review, Concept Analysis and Ways Forward, Journal of Clinical Nursing. (2016) 25, no. 9-10, 1464–1475, 10.1111/jocn.13180, 2-s2.0-84962564145. 26990036

[11] Cilluffo S. , Bassola B. , Pucciarelli G. , Vellone E. , and Lusignani M. , Mutuality in Nursing: a Conceptual Framework on the Relationship Between Patient and Nurse, Journal of Advanced Nursing. (2022) 78, no. 6, 1718–1730, 10.1111/jan.15129. 34873740

[12] Pucciarelli G. , Lyons K. S. , Simeone S. , Lee C. S. , Vellone E. , and Alvaro R. , Moderator Role of Mutuality on the Association Between Depression and Quality of Life in Stroke Survivor–Caregiver Dyads, Journal of Cardiovascular Nursing. (2021) 36, no. 3, 245–253, 10.1097/JCN.0000000000000728. 32740226

[13] Magasi S. , Buono S. , Yancy C. W. , Ramirez R. D. , and Grady K. L. , Preparedness and Mutuality Affect Quality of Life for Patients with Mechanical Circulatory Support and Their Caregivers, Circ Cardiovasc Qual Outcomes. (2019) 12, no. 1, 10.1161/CIRCOUTCOMES.117.004414, 2-s2.0-85059886937. 30636480

[14] Chen C. , Zhao Q. , Zhang X. et al., The Relationship Between Mutuality and Contributions to self-care in Family Caregivers of Patients with Heart Failure: Multiple Mediating Effects of Resilience and self-efficacy, European Journal of Cardiovascular Nursing. (2022) 21, no. 8, 812–820, 10.1093/eurjcn/zvac016. 35292823

[15] Vellone E. , Chung M. L. , Alvaro R. , Paturzo M. , and Dellafiore F. , The Influence of Mutuality on Self-Care in Heart Failure Patients and Caregivers: a Dyadic Analysis, Journal of Family Nursing. (2018) 24, no. 4, 563–584, 10.1177/1074840718809484, 2-s2.0-85058637855. 30453797

[16] Godwin K. M. , Swank P. R. , Vaeth P. , and Ostwald S. K. , The Longitudinal and Dyadic Effects of Mutuality on Perceived Stress for Stroke Survivors and Their Spousal Caregivers, Aging & Mental Health. (2013) 17, no. 4, 423–431, 10.1080/13607863.2012.756457, 2-s2.0-84876478576. 23323629 PMC3646563

[17] Dellafiore F. , Chung M. L. , Alvaro R. et al., The Association Between Mutuality, Anxiety, and Depression in Heart Failure Patient-Caregiver Dyads, Journal of Cardiovascular Nursing. (2019) 34, no. 6, 465–473, 10.1097/JCN.0000000000000599, 2-s2.0-85073183006. 31365444

[18] Cilluffo S. , Bassola B. , Pucciarelli G. et al., Mutuality Between Nurses and Patients with Chronic Illnesses: a Cross‐Sectional Descriptive Study, Scandinavian Journal of Caring Sciences. (2024) 38, no. 2, 487–495, 10.1111/scs.13251. 38459748

[19] Cilluffo S. , Lyons K. S. , Bassola B. et al., The Association Between Mutuality and Quality of Life in Adults with Chronic Illnesses and Their Nurses: Actor-Partner Interdependence Model Analysis, Journal of Clinical Nursing. (2025) 34, no. 8, 3288–3296, 10.1111/jocn.17510. 39468873

[20] Sandelowski M. , What’s in a Name? Qualitative Description Revisited, Research in Nursing & Health. (2010) 33, no. 1, 77–84, 10.1002/nur.20362, 2-s2.0-74349101245. 20014004

[21] Bradshaw C. , Atkinson S. , and Doody O. , Employing a Qualitative Description Approach in Health Care Research, Global Qualitative Nursing Research. (2017) 4, 10.1177/2333393617742282, 2-s2.0-85052202248. PMC5703087 29204457

[22] Doyle L. , McCabe C. , Keogh B. , Brady A. , and McCann M. , An Overview of the Qualitative Descriptive Design Within Nursing Research, Journal of Research in Nursing. (2020) 25, no. 5, 443–455, 10.1177/1744987119880234. 34394658 PMC7932381

[23] O’Brien B. C. , Harris I. B. , Beckman T. J. , Reed D. A. , and Cook D. A. , Standards for Reporting Qualitative Research, Academic Medicine. (2014) 89, no. 9, 1245–1251, 10.1097/ACM.0000000000000388, 2-s2.0-84908022244. 24979285

[24] Gale N. K. , Heath G. , Cameron E. , Rashid S. , and Redwood S. , Using the Framework Method for the Analysis of Qualitative Data in multi-disciplinary Health Research, BMC Medical Research Methodology. (2013) 13, no. 1, 10.1186/1471-2288-13-117, 2-s2.0-84884171494. PMC3848812 24047204

[25] Hennink M. M. , Kaiser B. N. , and Marconi V. C. , Code Saturation Versus Meaning Saturation, Qualitative Health Research. (2017) 27, no. 4, 591–608, 10.1177/1049732316665344, 2-s2.0-85013220243. 27670770 PMC9359070

[26] Lincoln Y. S. and Guba E. G. , But is it Rigorous? Trustworthiness and Authenticity in Naturalistic Evaluation, New Directions for Program Evaluation. (1986) 1986, no. 30, 73–84, 10.1002/ev.1427.

[27] McQueen A. , Nurse–Patient Relationships and Partnership in Hospital Care, Journal of Clinical Nursing. (2000) 9, no. 5, 723–731, 10.1046/j.1365-2702.2000.00424.x, 2-s2.0-0038096462.

[28] Feo R. , Rasmussen P. , Wiechula R. , Conroy T. , and Kitson A. , Developing Effective and Caring Nurse-Patient Relationships, Nursing Standard. (2017) 31, no. 28, 54–63, 10.7748/ns.2017.e10735, 2-s2.0-85047783205. 28271761

[29] Östman M. , Bäck‐Pettersson S. , Sundler A. J. , and Sandvik A. , Nurses’ Experiences of Continuity of Care for Patients with Heart Failure: A Thematic Analysis, Journal of Clinical Nursing. (2021) 30, no. 1-2, 276–286, 10.1111/jocn.15547. 33141466

[30] Kokorelias K. M. , Gignac M. A. M. , Naglie G. , and Cameron J. I. , Towards a Universal Model of Family Centered Care: A Scoping Review, BMC Health Services Research. (2019) 19, no. 1, 10.1186/s12913-019-4394-5, 2-s2.0-85070758139. PMC6693264 31409347

[31] Parmar J. , Anderson S. , Abbasi M. et al., Family Physician’s and Primary Care Team’s Perspectives on Supporting Family Caregivers in Primary Care Networks, International Journal of Environmental Research and Public Health. (2021) 18, no. 6, 10.3390/ijerph18063293. PMC8005195 33806725

[32] Wessel S. and Manthey M. , Primary Nursing: Person-Centered Care Delivery System Design, Minneapolis (MN): Creative Health Care Management. (2015) .

